# An outbreak of spotted fever rickettsiosis in U.S. Army troops deployed to Botswana.

**DOI:** 10.3201/eid0203.960309

**Published:** 1996

**Authors:** B L Smoak, J B McClain, J F Brundage, L Broadhurst, D J Kelly, G A Dasch, R N Miller

**Affiliations:** Division of Preventive Medicine, Walter Reed Army Institute of Research, Washington, D.C., USA.


### An Outbreak of Spotted Fever Rickettsiosis in U.S. Army Troops Deployed to Botswana

Novel infectious diseases are recognized when nonimmune persons move into the ecologic niche of a pathogen and become inadvertent hosts. One population at frequent risk to such emerging pathogens is the military. Historical examples of this phenomenon include outbreaks of scrub typhus during World War II and Korean hemorrhagic fever during the Korean War (1). In this report, we describe a modern example as we document an unusually high attack rate of a rickettsial disease among U.S. troops camping for a brief period in Africa.

In January 1992, U.S. soldiers participated in a 10-day training exercise with the Botswana Defense Force near Shoshong, Botswana. Activities included individual weapons' training and a series of mock battles. The terrain was generally flat with scattered bushes and small trees. The climate was semiarid, and temperatures reached 120°F during the day. During the fieldexercise, one soldier sought medical assistance to remove small "insects" crawling rapidly on his body. No specimens were reliably identified in the field or preserved for subsequent examination. No unusual illnesses were reported to medical personnel during the training exercise. After the field-training exercises, the soldiers spent 2 days in Gaborone, the capital of Botswana. Within 2 days of their return to their home station, approximately 30% of the deployed soldiers sought medical attention with symptoms of fever, headache, and regional lymphadenitis. Several soldiers reported insect bites. An epidemiologic team from Walter Reed Army Institute of Research was sent to Botswana to assist with the outbreak investigation.

From airplane manifests, 169 soldiers were identified as having been deployed to Botswana. One week after the soldiers returned to their home station, a questionnaire requesting information on symptoms, personal protective measures taken against arthropod vectors, reservoir exposures, and other potential risk factors for infection was administered to all available soldiers (n = 140); 132 soldiers underwent directed physical examinations, in which skin lesions, lymphadenopathy, and right upper quadrant tenderness were noted. Clothing and equipment used in the field that had not been cleaned since the exercise were visually examined in detail by a team of entomologists. Blood samples from 126 soldiers were obtained at 1 week and 10 weeks postdeployment. Serum specimens were divided into aliquots and frozen at -20°C for laboratory analyses.

Sera were tested for antibodies to rickettsiae by using an indirect fluorescence antibody technique (IFA) (2). Sera drawn at 10 weeks postdeployment were initially screened for whole immunoglobulin reactivity against *Rickettsia conorii* Moroccan strain, a member of the spotted fever group of rickettsiae that cause boutonneuse fever. Subsequently, sera showing titers ≥1:64 were paired with their respective 1-week samples and titered to the endpoint, defined as the highest dilution showing discernible fluorescing organisms. The rickettsial species used as antigens for antibody titration included cell culture–propagated *R. conorii* (Moroccan, ATCC VR-141), *Rickettsia akari* Hartford, and *Rickettsia typhi* (Wilmington, ATCC VR-144). The latter two microorganisms are the etiologic agents of rickettsial pox and murine typhus, respectively. Slides were read by one technician who was blinded to the clinical history corresponding to the sample.

A case-patient was defined as a soldier who had either a fourfold or greater rise in immunoglobulin G (IgG) titer to *R. conorii* within 9 weeks to a titer ≥1:128 by IFA, or clinically, by the presence of a *tache noire* (eschar) and at least two of the following symptoms: regional lymphadenitis, fever, chills, severe headache, and muscle or joint pains. By this case definition, the overall attack rate was 23% (39 of 169 soldiers). Of 36 case-patients who provided paired serum samples, 24 (67%) seroconverted during the postdeployment period. All 24 case-patients who seroconverted were symptomatic, but six did not have an eschar. Of the 15 case-patients without documented fourfold rises in titer, all had at least one eschar and regional lymphadenitis observed by a physician. Three of the soldiers who did not seroconvert, but were identified as case-patients by the clinical definition, had high standing titers, ≥1:256.

One week after returning from Botswana, the case-patients (n = 36) had a geometric mean reciprocal (GMT) IgG titer to *R. conorii* of 43, which increased to 185 9 weeks later (Student's *t* test paired, $p < 0.0001$). Over the same period, the GMT of non-case-patients (n = 38) who had positive titers on the initial whole immunoglobulin screening remained the same at 38. To determine whether early treatment impaired antibody production, the case-patients were divided into two groups: those who

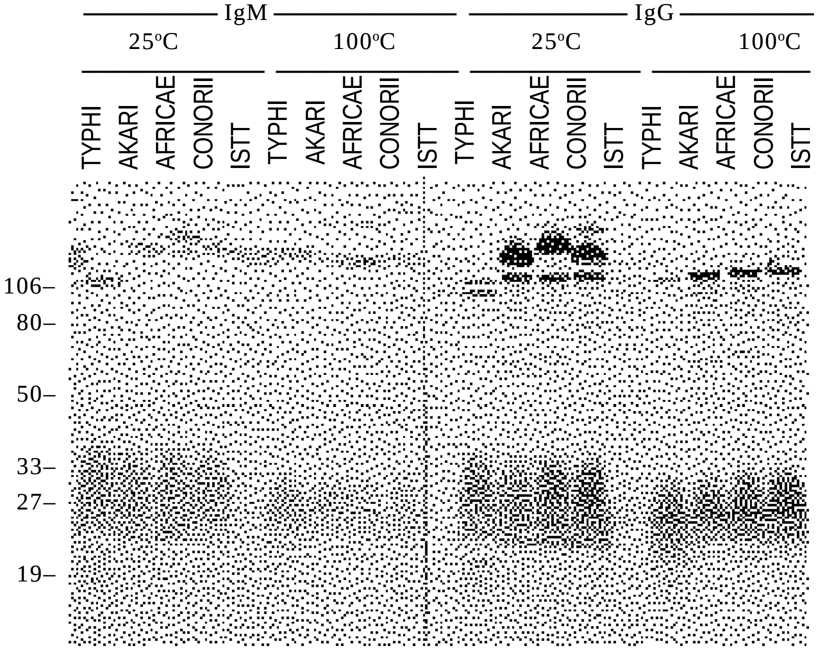

Figure 1. Western blot reactivity of convalescent-phase serum from a patient with spotted fever rickettsiosis with high standing titers. Antigens from the rickettsial isolates were solubilized at room temperature or boiled for 5 minutes before electrophoresis. The darkest large bands indicate *R. africae*, *R. conorii*, and Israeli tick typhus rickettsiae [ISTT] (no specificity detected). *R. typhi* is a member of the typhus group of rickettsiae, whereas all other isolates are members of the spotted fever group.

received treatment within 2 days of the start of their symptoms (n = 12) and those who received treatment 3 or more days later (n = 21). The group that was treated early had a lower mean GMT at follow-up, but the difference was not statistically significant (early 136, 95% confidence interval [C.I.]: 77, 239: late 197, 95% C.I.: 114, 338).

A Western blot immunoassay was used (3). Rickettsial antigens (*R. typhi* Wilmington, *R. akari*, *Rickettsia africae* F (4,5), *R. conorii* Malish (ATCC VR613), and Israeli tick typhus rickettsiae (T-487)) used in the immunoblots, either boiled or solubilized at room temperature, were electrophoresed on an 8% to 16% gradient PAGE gel. Renografin density gradient purified antigen was applied at 20 µg protein per lane. *R. typhi* was yolk sac–propagated, whereas spotted fever group antigens were grown in irradiated L cells. Patients' serum specimens were reacted with the electrophoresed antigens at 1:250 dilution. Horseradish peroxidase–labeled goat antibodies against human IgG (gamma-chain-specific) and IgM ( µ chain-specific) (Calbiochem, San Diego, CA) were used at 1:1000 dilution, and bound antibody was detected with 4-Cl-1-naphthol-$H_2O_2$ reagent. Prestained SDS-PAGE molecular weight standards (Bio-Rad, Melville, NY) were used to estimate antigen sizes

Western blots were used to characterize the serologic reactivities of 38 case-patients and 37 non–case-patients. Serum specimens were considered positive for reactivity with spotted fever rickettsial antigens if a characteristic broad washboard of spotted fever group lipopolysaccharide reactivity (SFG LPS) was observed in the 20- to 40-kDa region with anti-IgG conjugate (Figure 1). Most of these serum specimens also exhibited pronounced IgM reactivity, which sometimes included *R. typhi* LPS. Serologic specificity toward the individual spotted fever species was determined by the relative IgG reactivity to species-specific antigens (SPAs) solubilized at room temperature found at 125 to 145 kDa. Thirty-two of the 38 case-patient specimens tested by Western blot were positive for spotted fever group IgG LPS.

On Western blot, the sera of 23 case-patients reacted most strongly with *R. africae* SPA (not shown), one with *R. conorii* SPA, and eight showed no specificity (Figure 1). Nine of 37 soldiers who did not meet our case definition were positive for spotted fever group IgG LPS. The specimens of two reacted primarily with *R. africae* SPA, and specimens from the remaining seven had nonspecific patterns. Five of these nine soldiers reported no symptoms. Two soldiers reported a runny nose, another reported a rash on the dorsal portion of the right upper arm, and another had a tender raised papule. By Western blot criteria, these nine soldiers would be considered infected and may represent asymptomatic cases. However, they did not meet our case definition and were not counted as case-patients in our analyses.

Patients' sera showed expected broad cross-reactivity with other spotted fever group rickettsiae by IFA. In sera collected at 10 weeks, 17 (51%) of the case-patients had identical *R. akari* and *R. conorii* titers; a difference of one dilution in titers was observed in12 (36%) of the case-patients. The remaining cases had *R. akari* titers that were fourfold less than their *R. conorii* titer. However, no Western blots were positive for *R. akari*. All but two 10-week serum samples from the case-patients were nonreactive at a titer of <1:32 with *R. typhi*

antigen by IFA, and none were positive by Western blot. Serum from one case-patient had titers of 1:128 o *R. akari*, *R. typhi*, and *R. conorii*. Serum from the second case-patient had titers of 1:512 to *R. conorii* and 1:64 to *R. typhi*.

Median ages of the case-patients and non–case-patients were 23.5 and 22 years, respectively. Although a larger proportion of cases was found among the older age groups, this trend was not significant ($\chi^2$ trend, $p = 0.28$). Most of the soldiers (77%) were non-Hispanic white. There were too few soldiers in other race-ethnic groups to make separate comparisons, but when those from other ethnic backgrounds were combined into one group, the group had an attack rate (22%) similar to that of the non-Hispanic white soldiers (24%).

The illness observed in the soldiers was mild to moderately severe. Although all cases were symptomatic, some came to medical attention only through active case finding. Many case-patients had systemic complaints, commonly described as "flulike." Each of the classic symptoms associated with rickettsial diseases (i.e., fever, chills, headache, myalgia, and arthralgia) was reported by at least 59% of the case-patients but by fewer than 10% of the non–case-patients (Table 1). Gastrointestinal complaints of stomach pain, anorexia, nausea, or vomiting were also reported more frequently by the case-patients than by the non–case-patients. In contrast, diarrhea and upper respiratory symptoms were reported at similar rates among case-patients and non–case-patients. On physical examination, lymphadenitis was observed in 36 (92%) and right upper quadrant tenderness in 17 (44%) of the case-patients. The skin lesions began as a tender erythematous papule, which progressed over 24 to 36 hours to form one to three clear vesicles. The fragile vesicles ruptured, exposing an erythematous base, which darkened into a small eschar. The total duration of the evolution was 72 to 96 hours; 34 (87%) of case-patients had at least one eschar, and 10 of the case-patients had more than one. Of all eschars reported during physical examinations, 50% were located on the trunk; others were located on the head, neck, extremities, penis, and scrotum. No soldiers were hospitalized, and all case-patients responded rapidly to antibiotic treatment.

An incubation period of up to 6 days was estimated from the histories of the first two case-patients (Figure 2). The first case-patient reported onset of symptoms on January 20, 6 days after he arrived in Botswana as a member of an advance team. The second case-patient arrived in Botswana on January 18 and had onset of symptoms 6 days later. Thus, assuming a Botswana exposure, the time from infection to symptoms in these two case-patients could not have exceeded 6 days, an incubation time consistent with the 5- to 7-day period commonly reported for African spotted fever rickettsioses. Finally, since most case-patients reported symptoms during the 3 days before or the 4 days after leaving Botswana, we concluded that the period of high risk exposures occurred during field training in Shoshong and not in Gaborone.

Screening of clothing used during the field training found a crushed trombiculid mite on the boot of one of the case-patients. In addition, a live *Hyalomma marginatum* tick was found on parachute rigging used in Botswana. No rickettsiae were isolated from the tick or any human specimens.

No significant differences were found between case-patients and others in reported exposure to vectors, such as mosquitoes (68% vs. 62%; $\chi^2$, $p = 0.53$) and chiggers (19% vs 10%; $\chi^2$, $p = 0.14$). However, case-patients (68%) were more likely than non–case-patients (40%) to report tick bites ($\chi^2$, $p = 0.007$). Only one soldier reported finding an engorged tick on his body. Fewer than 50% of all deployed soldiers reported using a DEET-containing insect repellent at any time during the exercise, and fewer than 10% of the soldiers reported using permethrin (anacaricide and repellent applied to clothing). No statistical differences were found between case-patients and non–case patients in reported uses of these personal protective measures. Self-reported exposures of case-patients (n = 31) vs non-case-patients (n = 101) to potential reservoirs

Table 1. Symptoms of case-patients and non–case-patients during a spotted fever outbreak

| Symptoms | Case–patients; n=32(%) | Non–case-patients; n=107(%) | Risk ratio | 95% Confidence interval |
|---|---|---|---|---|
| Fever | 19 (59) | 8 (7) | 6.06 | 3.44,10.68 |
| Chills | 25 (78) | 6 (6) | 12.44 | 5.96,26.00 |
| Headache | 23 (72) | 8 (7) | 8.90 | 4.61,17.21 |
| Muscle ache | 26 (81) | 10 (9) | 12.40 | 5.56,27.66 |
| Joint pain | 21 (66) | 9 (8) | 6.94 | 3.78,12.73 |
| Fatigue | 27 (84) | 6 (6) | 17.35 | 7.26,41.43 |
| Lymphadenitis | 27 (84) | 3 (3) | 19.62 | 8.27,46.57 |
| Abdominal pain | 9 (28) | 4 (4) | 3.79 | 2.26,6.36 |
| Anorexia | 11 (34) | 3 (3) | 4.68 | 2.90,7.53 |
| Nausea/vomiting | 13 (41) | 3 (3) | 5.26 | 3.27,8.46 |
| Diarrhea | 7 (22) | 21 (20) | 1.11 | 0.54,2.30 |
| Cough | 4 (12) | 9 (8) | 1.38 | 0.58,3.33 |
| Nasal congestion | 7 (22) | 17 (16) | 1.34 | 0.66,2.74 |
| Sore throat | 6 (19) | 16 (15) | 1.23 | 0.57,2.63 |

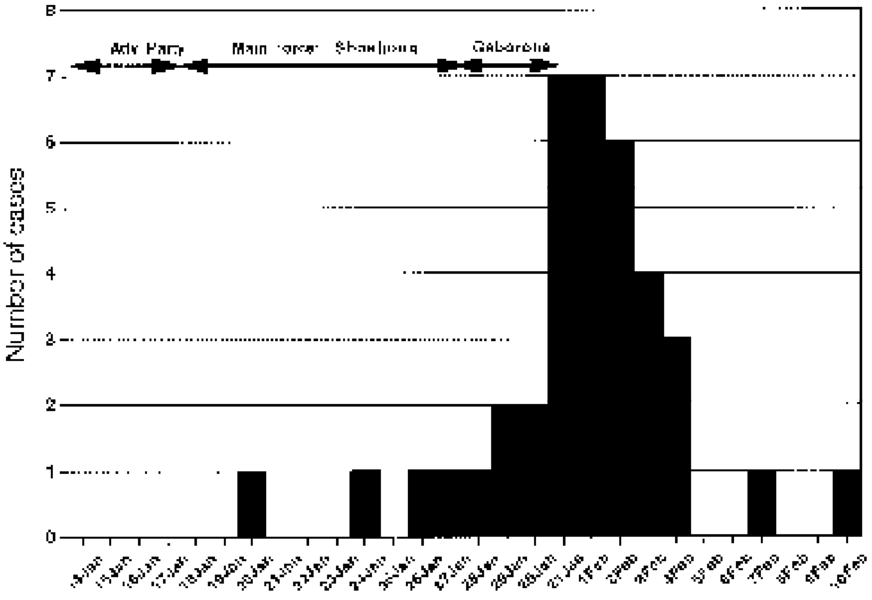

Figure 2. Epidemic curve of a spotted fever outbreak among U.S. troops.

of *R. conorii* (dogs: 52% vs 56%; rodents: 10% vs 35%; antelopes: 3% vs 7%; cattle: 81% vs 87%; horses:16% vs 25%; sheep: 42% vs 38%; and goats: 90% vs 87%) were not statistically different.

More than 85% (n = 117) of the soldiers slept on sleeping pads placed directly on the ground. Few (n = 18) soldiers reported sleeping on cots, litters, hammocks, or pallets. The attack rate was approximately twice as high among men sleeping on the ground (25%) as among those who did not (11%). Because of the small number who slept off the ground, this difference, was not statistically significant (risk ratio = 2.23; 95% C.I.: 0.58, 8.56). The attack rate among soldiers who maneuvered in the field during daily training exercises was lower than the rate among soldiers who remained at base camp (24% vs. 33%, respectively), but the difference was also not statistically significant (odds ratio = 1.40, 95% C.I.: 0.77, 2.56).

This report documents the occurrence of a large focal outbreak of a spotted fever group rickettsiosis among American soldiers participating in a short field-training exercise in Botswana. Little is known about the epidemiology of rickettsial diseases in Botswana. However, during the civil unrest that occurred in neighboring Rhodesia (now Zimbabwe) in the late 1970s, several thousand cases of tick typhus were reported among military personnel. In Zimbabwe, ticks removed from 100 soldiers were all larvae of *Amblyomma hebraeum* (6). In another study, 33% to 75% of *A. hebraeum* ticks, collected from animals or vegetation in four regions of Zimbabwe, were heavily infested with rickettsialike organisms (7). Only the recently characterized new rickettsia *R. africae*, but not *R. conorii*, the etiologic agent of boutonneuse fever, was detected in these ticks (4,7). In contrast, the dog ticks *Rhipicephalus simus* and *Haemaphysalis leachi* were less heavily infected (0% to 21%) and were only infected with *R. conorii*. *R. africae* has also been isolated from a hospitalized patient with fever, severe headache, and regional lymph-adenopathy but no rash other than an erythematous tick bite site(8).

On the other hand, in his original 1934 clinical description of African tick bite fever, Pijper (9) described a patient with a rash and eschar, whose condition he carefully distinguished from bouton-neuse fever. He clearly associated this milder disease with the "hardly visible" swarming larval veld ticks ("little pests") and not dog ticks. He also noted that even if the disease was not severe, it needed to be recognized by physicians and not confused with other potentially more devastating illnesses requiring more aggressive therapy.

Recent surveys of human antibodies in eight African countries have suggested a seroprevalence of antibodies to spotted fever rickettsiae of 0% to 52%, which parallels the distribution of *Amblyomma* ticks (10,11). The etiologic agent appeared to be *R. africae*, based on Western blot analysis (10). IFA analysis has been found inadequate in distinguishing human infections with *R. africae* and *R. conorii* (11).

The collective observations parallel our clinical, epidemiologic, and serologic findings on the Botswana outbreak and are most consistent with disease caused by *R. africae. R. africae* is closely related to *R. conorii* but has been proposed as a new species (5). Whether we consider this a newly emerging rickettsiosis or, in deference to the pioneering work of Pijper, a disease reemerging into our consciousness, African tick bite fever caused by *R. africae* may be epidemiologically distinct from urban *R. conorii* infections.

We have established the potential for high attack rates of this spotted fever rickettsiosis, but much more needs to be understood about the clinical severity of this disease and its potential for asymptomatic infections. Physically fit, healthy, young soldiers may not represent a particularly good model for assessing the severity of disease in populations residing in areas where the disease is endemic.

**Bonnie L. Smoak, M.D., Ph.D.*; J. Bruce McClain, M.D.‡; John F. Brundage, M.D.*; Laurel Broadhurst, M.D.*; Daryl J. Kelly, Ph.D.†§; Gregory A. Dasch, Ph.D.§; and Richard N. Miller, M.D.***

*Divisions of Preventive Medicine and †Communicable Diseases and Immunology, Walter Reed Army Institute of Research, Washington, D.C., USA; ‡U.S. Army MEDDAC, Vicenza, Italy; §Viral and Rickettsial Diseases Program, U.S. Naval Medical Research Institute, Bethesda, Maryland, USA

### Acknowledgment

This investigation was supported in part by the Naval Medical Research and Development Command, Research Task No. 61102A.010BJX.1293.

### References

1. Institute of Medicine. Emerging infections: microbial threats to health in the United States. Washington, DC: National Academy Press, 1992:110-2.
2. Philip RN, Casper EA, Burgdorfer W, et al.. Serologic typing of rickettsiae of the spotted fever group by microimmunofluorescence. J Clin Microbiol 1978;3:51-61.
3. Raoult D, Dasch GA. Line and Western blot immunoassays for diagnosis of Mediterranean spotted fever. J Clin Microbiol 1989;27:2073-9.
4. Kelly PJ, Beati L, Matthewman LA, Mason PR, Dasch GA, Raoult D. A new pathogenic spotted fever group rickettsia from Africa. J Trop Med Hyg 1994;97:129-37.
5. Kelly PJ, Beati L, Mason PR, Matthewman LA, Roux V, Raoult D. *Rickettsia africae* sp. nov., the etiological agent of African tick bite fever. Int J Syst Bacteriol 1996;46:611-4.
6. Yunker CE, Norval RAI. Observations on African tick typhus (tick-bite fever) in Zimbabwe. In: Fivaz B, Petney T, Horak I, editors. Tick vector biology: medical and veterinary aspects. Berlin: Springer Verlag 1989:143-7.
7. Beati L, Kelly PJ, Mattewman LA, Mason PR, Raoult D. Prevalence of rickettsia-like organisms and spotted fever group rickettsiae in ticks (Acari: Ixodidae) form Zimbabwe. J Med Entomol 1995;32:787-92.
8. Kelly P, Mattewman L, Beati L, Raoult D, Mason P, Dreary M, et al. African tick-bite fever: a new spotted fever group rickettsiosis under an old name. Lancet 1992;340:982-3.
9. Pijper A. Tick-bite fever: a clinical lecture. SA Med J 1934;8:551-6.
10. Dupont HT, Brouqui P, Faugere B, Raoult D. Prevalence of antibodies to *Coxiella burnetii, Rickettsia conorii*, and *Rickettsia typhi* in seven African countries. Clin Infect Dis 1995;21:1126-33.
11. Kelly PJ, Mason PR, Matthewman LA, Raoult D. Seroepidemiology of spotted fever group rickettsial infections in Zimbabwe. J Trop Med Hyg 1991;94:304-9.
